# Household-based cash transfer targeting strategies in Zimbabwe: Are we reaching the most vulnerable children?

**DOI:** 10.1016/j.socscimed.2012.09.031

**Published:** 2012-12

**Authors:** Laura Robertson, Phyllis Mushati, Jeffrey W. Eaton, Lorraine Sherr, Jeremiah C. Makoni, Morten Skovdal, Tom Crea, Gideon Mavise, Lovemore Dumba, Christina Schumacher, Shungu Munyati, Constance Nyamukapa, Simon Gregson

**Affiliations:** a Department of Infectious Disease Epidemiology, School of Public Health, Imperial College London, St Mary's Campus, Norfolk Place, London W2 1PG, UK; b Biomedical Research & Training Institute, Harare, Zimbabwe; c Department of Infection and Population Health, University College London, UK; d DOMCCP, Manicaland, Zimbabwe; e Department of Health Promotion and Development, University of Bergen, Norway; f Graduate School of Social Work, Boston College, USA; g Catholic Relief Services, Harare, Zimbabwe; h Department of Pediatrics, Johns Hopkins University School of Medicine, Baltimore, MD, USA

**Keywords:** Sub-Saharan Africa, Zimbabwe, Children, Cash transfers, HIV/AIDS, Social welfare

## Abstract

Census data, collected in July 2009, from 27,672 children were used to compare the effectiveness, coverage and efficacy of three household-based methods for targeting cash transfers to vulnerable children in eastern Zimbabwe: targeting the poorest households using a wealth index; targeting HIV-affected households using socio-demographic information (households caring for orphans, chronically-ill or disabled members; child-headed households); and targeting labour-constrained households using dependency ratios. All three methods failed to identify large numbers of children with poor social and educational outcomes. The wealth index approach was the most efficient at reaching children with poor outcomes whilst socio-demographic targeting reached more vulnerable children but was less efficient.

## Highlights

► We compared three methods for targeting cash transfer programmes to vulnerable children in Manicaland, Zimbabwe. ► All the methods failed to identify large numbers of children with poor health and education outcomes. ► Targeting poor children using an asset-based wealth index was the most efficient method for reaching vulnerable children. ► Targeting based on socio-demographic criteria improved coverage of vulnerable children but was less efficient.

## Introduction

Cash transfer programmes have become popular social welfare interventions in low- and middle-income countries (Shibuya, 2008) due to their success at improving child health and education outcomes (Adato & Bassett, 2009). Evidence on the relative effectiveness of alternative targeting strategies is needed to ensure that these programmes reach the most vulnerable children, particularly in countries in sub-Saharan Africa subject to generalised HIV epidemics and widespread poverty.

Established national programmes in Latin America (e.g. Mexico (Skoufias, Davis, & Behrman, 1999)) target children living in the poorest households using routinely collected data on household income. In African countries, these data are less available and several programmes (e.g. in Zambia (MCDSS & GTZ, 2006) and Malawi (Miller, Tsoka, & Reichert, 2008)) have targeted “ultra-poor, labour-constrained households” using community committees and survey data to identify households with high ratios of dependents (children, elderly and sick adults) to working-age adults. This method is simple and is designed to reach households that are economically vulnerable or suffering from the demographic consequences of the HIV epidemic (i.e. the illness and death of working-age adults (MOHCW, 2010)). In Zambia, targeted households were more likely to be elderly or single-headed or to contain orphans or disabled members (MCDSS & GTZ, 2006). In Malawi, targeted households were more likely to be caring for orphans or someone sick with HIV or TB (Miller et al., 2008). However, these demographic characteristics are endogenously related to the definition of a labour-constrained household and rigorous evaluations are needed to establish whether targeting labour-constrained households actually works in identifying the most vulnerable children and to compare the method with other household-based and non-household-based approaches.

In this paper, we use data from the baseline census conducted for a community-randomised controlled trial of a cash transfer programme in Manicaland, eastern Zimbabwe to compare the effectiveness, coverage and efficiency, with respect to reaching children with poor outcomes, of three alternative household-based targeting methods that have been used by cash transfer programmes in sub-Saharan Africa: (1) targeting the poorest households using a wealth index; (2) targeting HIV-affected households using socio-demographic information; and (3) targeting labour-constrained households using high dependency ratios.

## Data and methods

This study was conducted using data collected in the baseline census for a community-randomised controlled trial of the effects of cash transfers for OVC on birth registration, vaccination coverage, and school attendance, in Manicaland province, eastern Zimbabwe (Robertson, Nyamukapa, Mushati, Munyati, & Gregson, 2011). As in the rest of the country, the study communities have been subject to a major HIV epidemic (in Zimbabwe as a whole, in 2009), there were 1.2 million people living with HIV and 84,000 deaths due to AIDS in a population of approximately 12 million (MOHCW, 2010) and have suffered the effects of a severe economic decline culminating in the collapse of the local currency in 2009. These factors have increased the numbers of orphans and other vulnerable children (OVC) in the country in need of assistance through a combination of extreme poverty and HIV-related illness, death and stigma within families and communities (Richter, 2010).

The baseline census for the Manicaland cash transfer trial was conducted in July 2009 in 30 communities representing four socio-economic strata (small towns, agricultural estates, roadside settlements and subsistence farming areas). Lists of all households in these communities were compiled from records of households that had ever been enumerated as part of an ongoing cohort study in the area, which has performed a census every two or three years since 1998 (Gregson et al., 2006). New households were added to the list as they were encountered. Local guides asked representatives from households in their area to attend at central meeting points on one of up to three different days. Trained research assistants conducted census interviews, in the local language *Shona*, with these household representatives. Ethical approval for the trial was granted by Imperial College Research Ethics Committee (ICREC_9_3_10), Biomedical Research and Training Institute's Institutional Review Board (AP81/09), and Medical Research Council of Zimbabwe (MRCZ/A/1518).

In the Manicaland cash transfer trial, following consultations with the local community and other key stakeholders undertaken in a preliminary feasibility study, it was decided to target the poorest 20% of households and those affected by HIV/AIDS (Robertson et al., 2011). For the current analysis, we used data from the baseline census to identify the households that would have been included in the programme if three alternative strategies for targeting vulnerable children had been applied – (1) targeting the poorest 20% of households; (2) targeting based on HIV related socio-demographic vulnerability; and (3) targeting labour-constrained households.

Identifying the poorest households within a community requires a measure of household wealth. Ideally estimates would be based on data on household income or expenditure (Howe, Hargreaves, & Huttly, 2008). However, in the predominantly rural communities in Manicaland, as in much of Africa, many households subsist outside of the cash economy. In low-income countries, information on household assets is often used to create an index of household wealth which can be used to rank the households within a population according to their relative wealth and identify the poorest households (Howe et al., 2008). In the Manicaland study, a wealth index was created for all households in the study areas using a simple summed score of household asset ownership based on the following assets (Lopman et al., 2007): source of drinking water, access to electricity, type of toilet facility, type of house, type of floor in the main dwelling, ownership of a radio, a television, a motorbike or a car. In each community in the study, the households were ranked using this index and the poorest 20% were identified.

In the trial feasibility study, a vulnerability mapping exercise found a number of household-level HIV-related socio-demographic characteristics to be predictors of poor child outcomes – caring for orphans (<18 years), chronic illness and disability amongst household members, and child (<18 years)-headed households. In this analysis, households with one or more of these characteristics were treated as socio-demographically vulnerable (further information on the definitions used for these variables is available in the Supplementary material).

Labour-constrained households were defined as those with no able-bodied adult (aged 19–64 years) or with a dependency ratio greater than 3. Dependency ratios were calculated by dividing the number of dependents in the household – those under 19 years, aged 65 years and above, chronically ill and disabled household members – by the number of healthy, able-bodied adults (MCDSS & GTZ, 2006).

In the baseline census, we also collected data on four direct indicators of child vulnerability, originally for use as primary endpoint indicators for the cash transfer trial: lack of a birth certificate amongst children aged 0–4 years; incomplete vaccination record amongst children aged 0–4 years; and poor school attendance amongst children aged 6–12 years and 13–17 years. A child was defined as having poor school attendance if they were not enrolled in school or if they were enrolled but attended less than 80% of days in the last 20 school days. An incomplete vaccination record was defined as not having received vaccination for BGC, measles, polio, or diphtheria by the appropriate age. In the current analysis, these outcomes were used as direct indicators of child vulnerability (poor outcomes) for comparing the effectiveness, coverage and efficiency of the three household-level targeting strategies in reaching vulnerable children.

To investigate the effectiveness of the targeting methods, we used age- and sex-adjusted logistic regression models to compare the probability that children in targeted and not-targeted households had poor outcomes for each of the proposed methods. To estimate the coverage of the different targeting methods, the extent to which children with poor outcomes were excluded and children with good outcomes (i.e. not poor outcomes) were included, we present the proportion of children with poor outcomes, with good outcomes and all children that were reached by each of the methods. We compared the efficiency of the three targeting methods by calculating the number of children with each of the poor outcomes that were reached per child targeted.

Finally, in addition to each targeting strategy separately, targeting strategies were considered in the following combinations: (1) targeting the poorest 20% of households; (2) targeting the poorest households plus those caring for orphans and child-headed households; and (3) targeting the poorest households, those caring for orphans and child-headed households plus those with chronically-ill or disabled members.

## Results

In the cohort survey in Manicaland, 16,887 households had been enumerated in at least one census since 1998, of which 11,820 households (70%) completed a cash transfer trial baseline census. Of those households not completing a baseline census, 10 (0.06%) declined to be interviewed, 2358 (14%) had relocated, for 1836 (11%) their dwelling was empty or no longer existed, and for 863 (5%) the reason they were not interviewed was unknown. 10,536 (89%) of households that completed the census reported caring for at least one child.

There were 27,672 children aged 0–17 years enumerated in the baseline census as regular residents of households in the study area. Of these, 5112 (19%) were identified as living in households in the lowest wealth quintile of the wealth index, 18,062 (67%) were targeted by the HIV-related socio-demographic criteria (households caring for orphans, chronically ill or disabled household members, or child-headed households), and 9013 (33%) were living in labour-constrained households (for information on the degree of overlap between the different targeting methods, see the Supplementary material). Amongst children aged 0–4 years, 6309 (54%) did not have a birth certificate and 5772 (36%) had an incomplete vaccination record. Amongst children aged 6–12 years and 13–17 years, 11,203 (21%) and 7837 (28%) had poor school attendance, respectively.

### Comparing the effectiveness of the targeting methods at reaching children with poor outcomes

The asset-based wealth index reached children who were significantly more likely to lack a birth certificate than non-targeted children (AOR 1.68, 95% CI 1.47–1.90, Table 1). Children targeted in socio-demographically vulnerable and labour-constrained households were also more likely to lack a birth certificate but the effect sizes were smaller and were of borderline statistical significance (AOR 1.10, 0.99–1.21 and AOR 1.14, 1.01–1.29, respectively). All three targeting methods reached children 6–12 years and 13–17 years who were significantly more likely to have poor school attendance than non-targeted children. Amongst children 6–12 years, the asset-based wealth index (AOR 1.51, 1.35–1.68), socio-demographic vulnerability targeting (AOR 1.51, 1.36–1.69), and dependency ratio targeting (AOR 1.46, 1.33–1.60) were equally effective at targeting children with poor attendance. Amongst children aged 13–17 years, the effect sizes were largest for the wealth index method (AOR 1.89, 1.67–2.15) and smallest for targeting based on dependency ratios (AOR 1.52, 1.37–1.68). None of the methods was successful in targeting children with incomplete vaccination records. This may be due to the presence of mobile vaccination units that operate in Manicaland and which may be effective in reaching children regardless of their poverty or vulnerability characteristics.

### Comparing the coverage and efficiency of the targeting methods in reaching vulnerable children

**Fig. 1:**
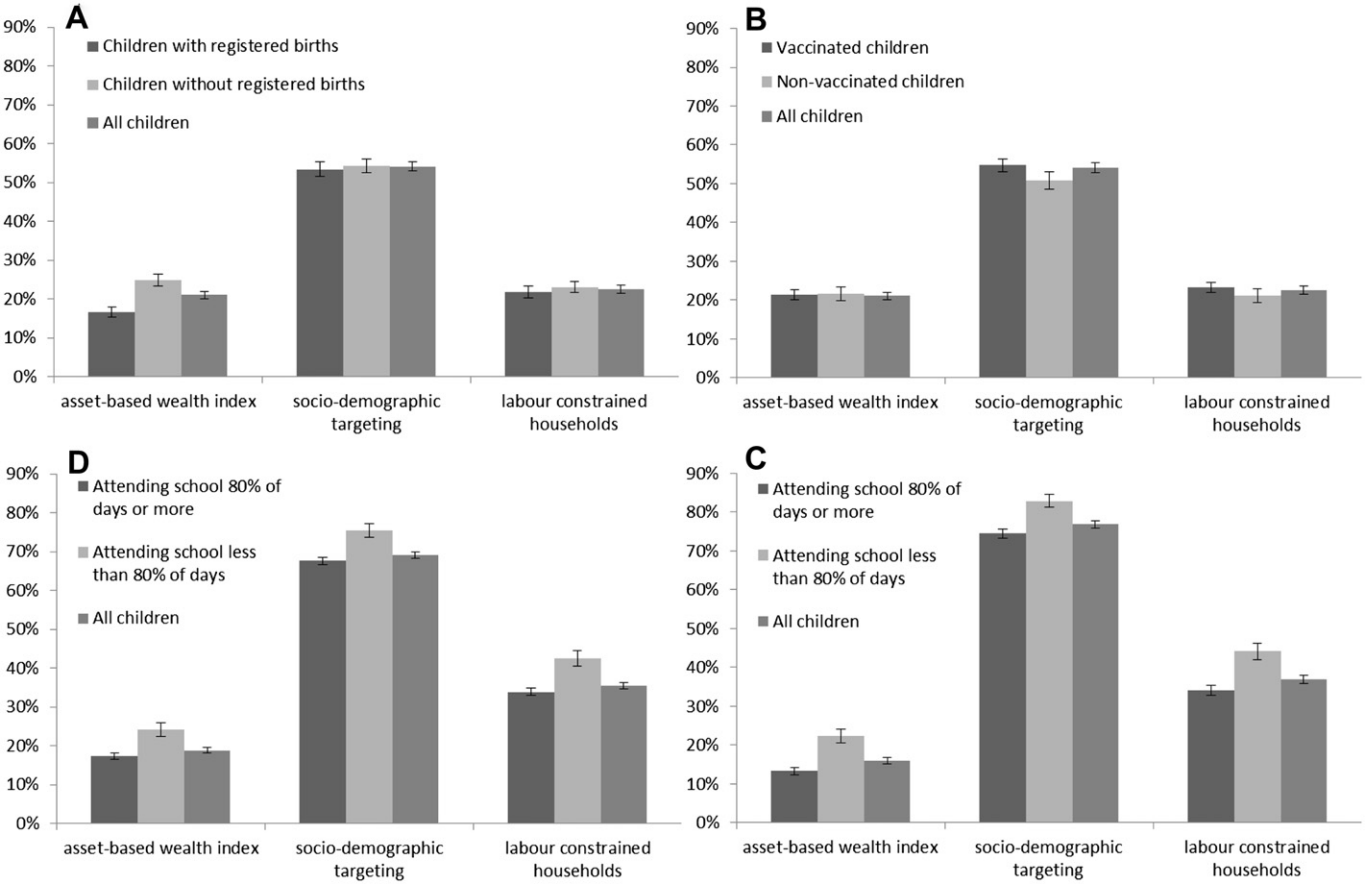
Comparison of the proportions of children with good outcomes, poor outcomes and all children who are targeted for each of three targeting methods – A: Percentage of children aged 0–4 years targeted by birth registration status, B: Percentage of children 0–4 years targeted by vaccination status, C: Percentage of children aged 6–12 years targeted by level of school attendance, D: Percentage of children aged 13–17 years targeted by level of school attendance.

Fig. 1 compares the proportions of children with good outcomes, of children with poor outcomes, and of all children targeted by the different targeting methods. Coverage of children with each of the poor outcomes was low (less than 50% in most cases) for all three targeting methods. Socio-demographic targeting reached a greater proportion of vulnerable children than the poverty-based measures; although, for the wealth index, the definition of poorest households could be broadened (e.g. from 20% to 40%) to increase the numbers of children reached.

Children without a birth certificate had significantly higher levels of coverage, when the wealth index was used for targeting, than children in the general population as a whole but there were no differences in coverage when the socio-demographic and labour-constrained household methods of targeting were applied (panel A). Children with poor school attendance had significantly higher levels of coverage with each of the three targeting methods than children in the general population (panel C & D).

In many cases, children with good outcomes had similar levels of coverage to children in the general population. However, children aged 0–4 years who had a birth certificate and children aged 6–12 years with good school attendance had significantly lower coverage when targeting was done using the wealth index. Children aged 13–17 years with good school attendance had significantly lower coverage than children as a whole for all three targeting methods.

Table 1 also compares the number of children with each poor outcome that were reached per child targeted (a measure of efficiency) for each of the targeting methods. In all cases, this number was below two thirds, which suggests that large numbers of children who do not suffer from these poor outcomes were included. The wealth index was more efficient at targeting vulnerable children than the methods based on socio-demographic vulnerability or labour-constrained households for each of the poor outcomes. These differences were statistically significant for birth registration amongst children aged 0–4 years and for school attendance amongst children aged 13–17 years.

### What happens when we combine different targeting strategies?

**Fig. 2:**
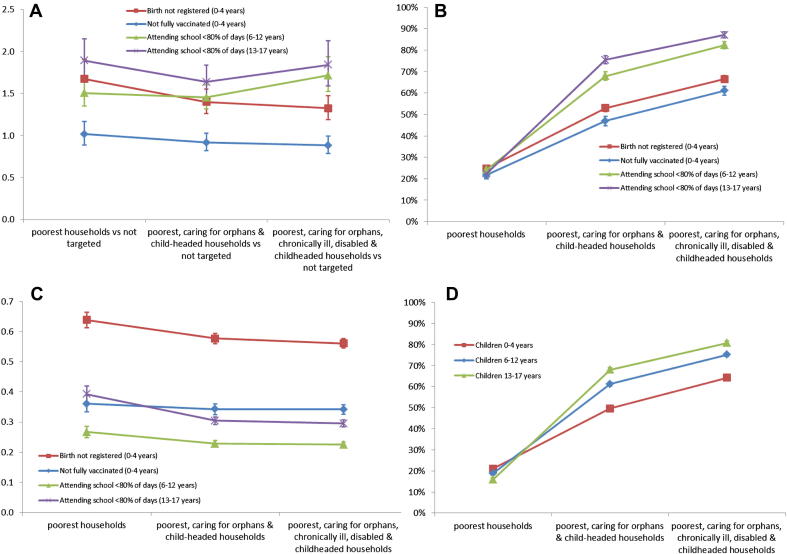
The effect of incremental increases in the number of targeting criteria used on the effectiveness, coverage and efficiency of the targeting strategy in reaching vulnerable children: A: Age- and sex-adjusted odds ratios comparing the likelihood of poor outcomes amongst targeted and non-targeted children, B: Proportion of children with poor outcomes targeted, C: Number of children with poor outcomes per child targeted, D: Proportion of all children targeted.

Fig. 2 examines the effects of incremental increases in the number of targeting criteria used to identify vulnerable children. For birth registration, as the number of targeting methods used in combination was increased, targeted children aged 0–4 years became increasingly less likely to be without a birth certificate compared to non-targeted children (panel A). However, the proportion of children without a birth certificate that were reached increased as more targeting criteria were added (panel B). The efficiency of the targeting strategies in targeting children without a birth certificate fell somewhat as more targeting criteria were included (panel C) so the improved coverage of vulnerable children resulted from greater inclusion of all children aged 0–4 years in the population as more targeting methods were added (panel D).

For school attendance amongst children aged 6–12 years, the odds ratios comparing targeted and non-targeted children fell slightly as the targeting criteria were expanded from just the poorest households to include child-headed households and those caring for orphans (panel A). When the targeting criteria were expanded further to include households caring for chronically-ill and disabled members, targeted children became more likely to have poor school attendance compared to non-targeted children, although this change was not statistically significant. The proportion of all children aged 6–12 years reached (panel D) and the proportion of children with poor attendance that were reached (panel B) increased as the number of targeting methods used increased, with the sharpest increase occurring when targeting the poorest households was combined with households caring for orphans and child-headed households. The number of children aged 6–12 years with poor school attendance that were reached per child targeted (efficiency) fell slightly as the targeting criteria were expanded to include households caring for orphans and child-headed households but remained constant when households caring for chronically-ill and disabled members were included (panel C).

The results were similar for school attendance amongst children aged 13–17 years. When households caring for orphans and child-headed households were targeted alongside the poorest households, targeted children became less likely to have poor school attendance, although this change was not statistically significant (panel A). When households caring for chronically-ill and disabled members were included, the odds ratio increased further, although, again, the change was not statistically significant. The proportion of children aged 13–17 years as a whole (panel D) and the proportion of children with poor school attendance (panel B) that were reached increased as the number of targeting methods increased, with the steepest increase again occurring when targeting the poorest households was combined with targeting households caring for orphans and child-headed households. The number of children aged 13–17 years with poor school attendance that were reached per child targeted decreased quite sharply as households caring for orphans and child-headed households were included in the targeting method but remained constant when households caring for chronically-ill and disabled members were included (panel C).

## Discussion

In this paper, we compared the effectiveness, coverage, and efficiency of three methods for targeting cash transfer programmes to children with poor health, education and other development outcomes. All of the methods effectively reached children at increased risk of poor school attendance and covered higher proportions of children with poor school attendance than children in the population as a whole. The wealth index method also effectively targeted children lacking birth certificates and covered a greater proportion of these children compared with children in the general population. However, none of the methods was effective in targeting children who were behind with their vaccinations and none was successful in achieving high coverage (large proportions of children with poor outcomes were missed) or efficiency (many apparently less vulnerable children were included).

Compared to the other methods, the asset-based wealth index was the most effective and efficient in targeting children with poor development and education outcomes. Targeting based on HIV-related socio-demographic vulnerability increased the coverage of vulnerable children but at the price of a decrease in efficiency – more children needed to be targeted overall to reach similar proportions of children with poor outcomes. Targeting children in labour-constrained households produced similar levels of coverage of vulnerable children to the asset-based wealth index but in a less efficient manner. Combining the asset-based wealth index method with targeting based on HIV-related socio-demographic vulnerabilities improved coverage amongst children with poor outcomes with generally modest reductions in efficiency.

The conclusions that can be drawn from these findings will depend on the type of social welfare intervention being considered and the priorities of those delivering it. If the priority is to target as many vulnerable children as possible, then targeting based on socio-demographic vulnerability will reach a large number of disadvantaged children. However, if, as is usually the case, resources are limited, a more efficient method with lower coverage, such as targeting a defined percentage of the poorest children, may be preferred.

The coverage of the wealth index-based targeting method could be increased by raising the threshold for inclusion (e.g. from the poorest 20% to the poorest 40%). Findings from an agricultural intervention in Malawi suggest that, in communities where poverty is widespread, targeting a small percentage of households can be perceived as unfair leading to resentment and conflict (Levy & Barahona, 2002). In the cash transfer programme in Manicaland, the community were consulted and closely involved in the design and implementation of the programme and qualitative data suggest that the programme, including the targeting, was socially acceptable and did not lead to conflict (Skovdal et al., submitted for publication).

The current study did not collect data for measuring and comparing the cost-effectiveness of the different targeting methods. However, such analyses are also important for the design of targeting strategies for cash transfer programmes. In some circumstances, the cost of implementing a narrow targeting strategy could be disproportionate compared to the cost of reaching all, or a larger proportion, of households in a community (Levy & Barahona, 2002). However, where gains in coverage are offset by losses in efficiency, cost-effectiveness analyses may be helpful in identifying the optimal targeting strategy.

It is a concern that all three of the household-based targeting methods examined here would have missed a large proportion of children with poor outcomes. In this context, targeting methods that achieve greater coverage may be preferable to those with higher efficiency, particularly since the differences in efficiency between the methods were relatively small. Further work is needed to improve the targeting of cash transfer programmes to the most disadvantaged children. However, it should be noted that the child outcomes considered here are caused by a variety of factors. Identifying a small number of specific characteristics that predict these outcomes well will be challenging for any potential targeting method.

In this study, we compared three strategies that target vulnerable children based on household characteristics. None of these strategies was successful in achieving high coverage or efficiency in reaching vulnerable children so alternative methods may need to be considered. Child characteristics may be better predictors of poor outcomes and, especially for interventions designed to improve a particular set of outcomes; it may be preferable to target children with these poor outcomes directly (e.g. children not in school). This would improve the efficiency of the targeting but targeting certain children within households can introduce conflict and could undermine the effectiveness of the intervention. A possible solution to this may be to target assistance to households with poor child outcomes (e.g. households with at least one child out of school) – rather than to the children directly – or to use a combination of household and child characteristics in selecting eligible households. Further work is required to investigate these forms of targeting. Reaching children outside the household is another possible alternative. School-based support for OVC has become increasingly popular in recent years with interventions to address problems ranging from micronutrient deficiencies to HIV prevention (PCD, 2012). However, in settings where not all children attend school, household-based programmes may still be needed.

Targeting children in labour-constrained households is used currently in several cash transfer programmes in sub-Saharan Africa. The method has been evaluated previously by investigating the frequency of inclusion and exclusion errors (MCDSS & GTZ, 2006; Miller et al., 2008). However, the findings presented here are of concern since they suggest that the method misses many vulnerable children and may be less efficient than alternative household-based methods. Current assistance for OVC in Manicaland most often is targeted to socio-demographically vulnerable and labour-constrained households rather than to the poorest households, who care for the most vulnerable children (see Supplementary material). Our findings suggest that policymakers and service providers may need to reconsider their perceptions about which households and children are the most vulnerable. At the same time, more research is needed to expand the evidence base on the performance of alternative targeting strategies.

Our baseline dataset was large and represents several socio-demographic locations so the findings may be generalizable to similar rural settings elsewhere in sub-Saharan Africa. A limitation of the study is that the data are cross-sectional. In the future, longitudinal data could be used to identify characteristics that are causally linked, over time, to poor outcomes. These characteristics could be useful indicators for targeting interventions to prevent poor outcomes occurring in the first place.

This paper compares the effectiveness, coverage and efficiency of alternative household-based targeting methods at reaching children with poor health, education and development outcomes using data from a household census. In predominantly rural areas, where few children stay in institutions or on the street, targeting methods that rely on data collected in a household census can be effective in ensuring that all households are considered and thereby can contribute to limiting omissions of vulnerable children. However, the data may be subject to reporting bias and standard household census procedures, on their own, do not provide opportunities for community involvement in identifying vulnerable households. Community-based participatory approaches drawing on local knowledge about local household and children's vulnerability have been used in targeting social welfare programmes (Pronyk et al., 2006) and may increase local ownership of these programmes but these approaches require further evaluation.

## Figures and Tables

**Table 1:** Comparison of the effectiveness and efficiency of three household-based targeting methods: (1) age- and sex-adjusted odds ratios comparing the likelihood of poor outcomes amongst targeted and non-targeted children; and (2) the number of children with each poor outcome reached per child targeted.

	Asset-based wealth index			Socio-demographic targeting			Labour constrained households		
AOR	95% CI	*N*	AOR	95% CI	*N*	AOR	95% CI	*N*
*(1) Age- and sex-adjusted comparisons of targeted and non-targeted children*									
Birth not registered (0–4 years)	1.68	1.47–1.90	6217	1.10	0.99–1.21	6121	1.14	1.01–1.29	6261
Not fully vaccinated (0–4 years)	1.02	0.89–1.17	5697	0.93	0.83–1.04	5608	0.95	0.83–1.09	5732
Attending school less than 80% of days (6–12 years)	1.51	1.35–1.68	11,098	1.51	1.36–1.69	10,784	1.46	1.33–1.60	11,173
Attending school less than 80% of days (13–17 years)	1.89	1.67–2.15	7766	1.63	1.43–1.85	7564	1.52	1.37–1.68	7824

	Asset-based wealth index			Socio-demographic targeting			Labour constrained households		
Number of children with poor outcomes reached per child targeted	95% CI	*N*	Number of children with poor outcomes reached per child targeted	95% CI	*N*	Number of children with poor outcomes reached per child targeted	95% CI	*N*
*(2) Number of children with poor outcomes reached per child targeted*									
Birth not registered (0–4 years)	0.64	0.61–0.66	1324	0.54	0.53–0.56	3329	0.55	0.53–0.58	1422
Not fully vaccinated (0–4 years)	0.36	0.33–0.39	1233	0.34	0.32–0.36	3011	0.33	0.31–0.36	1300
Attending school less than 80% of days (6–12 years)	0.27	0.25–0.29	2094	0.22	0.21–0.23	7487	0.25	0.23–0.26	3999
Attending school less than 80% of days (13–17 years)	0.39	0.36–0.42	1229	0.30	0.28–0.31	5819	0.33	0.31–0.35	2887
