# Supplementary material for: Household-based cash transfer targeting strategies in Zimbabwe: Are we reaching the most vulnerable children?
Source: Soc Sci Med. 2012 Dec;75(12):2503–8. doi: 10.1016/j.socscimed.2012.09.031 (PMC3512055; doi:10.1016/j.socscimed.2012.09.031)
Supplement: Supplementary file 1 [file mmc1.docx]

**Online supplementary material: Cash transfer targeting strategies in Zimbabwe: are we reaching the most vulnerable children?**

**Data definitions**

The following definitions were used throughout the paper:

Chronic illness – defined as very sick for at least 3 months during the past 12 months, where “very sick” is defined as being too sick to work or do normal activities around the house.

Disability – respondents were asked to report for each household member whether they had any form of disability. Answer options included blind or visually impaired, deaf, mute, mental illness, physical disability or other.

Vaccinations – a child aged 0-4 years was defined as having an up-to-date vaccination record if they had received their BCG, measles, DPT and polio vaccinations by the appropriate age. In Zimbabwe, the BCG vaccination is usually given at birth or within the first four months of life. The measles vaccine should be given within the first nine months of life. The DPT and polio vaccinations should both be given in three doses during the 4^th^, 5^th^ and 6^th^ months of life.

**Degree of overlap between different targeting methods at the household level**

We constructed a Venn diagram (figure 1) to compare the degree of overlap, at the household level, between each of the three targeting strategies: (1) targeting children living in the poorest 20% of households as identified by the asset-based wealth index; (2) targeting based on HIV-related, socio-demographic vulnerability (i.e. households caring for orphans, chronically ill or disabled household members and child-headed households); and (3) targeting labour constrained households. Only households that reported that they cared for at least one child were included in this analysis.

It is clear from figure 1 that there is a large degree of overlap between the different targeting methods. If we targeted socio-demographically vulnerable households, we would also be targeting 92% of labour constrained households and 60% of the poorest households. However, targeting labour constrained households would only reach 39% of socio-demographically vulnerable households and 27% of the poorest households. Targeting poor households using the asset-based wealth index would target 18% of labour constrained households and 17% of socio-demographically vulnerable households, which suggests that targeting based on the lowest quintile of the index does not target a higher proportion of labour constrained or socio-demographically vulnerable households.

***Figure 1: Venn diagram of overlap between different targeting methods***

POOR HOUSEHOLDS (WEALTH INDEX)

LABOUR CONSTRAINED

HIV-RELATED, SOCIO-DEMOGRAPHIC TARGETING

191

(2.6%)

685

(9.4%)

3,248

(44.8%)

630

(8.7%)

2,014

(27.8%)

33

(0.5%)

455

(6.3%)

Total households with non-missing data on all targeting methods: 10,177

Total households eligible under at least one targeting method: 7,256

Total households eligible under none of the targeting methods: 2,921

**Current assistance for OVC in Manicaland, Zimbabwe**

During the baseline census of the Manicaland Cash Transfer Trial, data were collected on the current level of assistance that households received. Only 7% (780) of the 10,536 households that completed the census and reported caring for at least one child reported currently receiving some form of external assistance. Labour constrained and socio-demographically vulnerable households were significantly more likely to be receiving assistance (10% vs. 3%; p<0.001 and 10% vs. 6%; p<0.001 respectively). Households in the poorest wealth quintile according to the asset-based wealth index were found to be significantly less likely to be receiving external assistance (6% vs. 8%; p=0.002). Amongst those receiving assistance, the most common forms of external assistance were payment of school fees (27%), food assistance (48%) and clothes or other supplies (13%). Very few reported cash assistance (2%) or psychosocial support (1%).
